# In vitro ultrastructure and biodegradation of activated plasma albumin gel derived from human samples: A prospective observational study

**DOI:** 10.1002/cap.10330

**Published:** 2024-12-18

**Authors:** Behzad Houshmand, Mohammadreza Talebi Ardakani, Farshad Armandei, Anahita Moscowchi, Ahmad Nazari, Jafar Ai, Mehdi Ekhlasmand Kermani, Hamoun Sabri

**Affiliations:** ^1^ Department of Periodontics, School of Dentistry Shahid Beheshti University of Medical Sciences Tehran Iran; ^2^ Aria Vira Academy Tehran Iran; ^3^ Department of Tissue Engineering, School of Advanced Technologies in Medicine Tehran University of Medical Sciences Tehran Iran; ^4^ School of Dentistry Kerman University of Medical Sciences Kerman Iran; ^5^ Department of Periodontics and Oral Medicine University of Michigan School of Dentistry Ann Arbor Michigan USA

**Keywords:** connective tissue, guided tissue regeneration, platelet‐rich fibrin, platelet‐rich plasma

## Abstract

**Background:**

In soft tissue regeneration, the clinical efficacy of fibrin membranes has been a pressing concern. The key to this efficacy lies in the stability of membrane and its controlled absorption. Human serum albumin, with its influence on the formation and stability of fibrin networks, could hold the key to developing a more stable alternative. This study investigates the ultrastructure and biodegradability of plasma albumin‐activated gel, a potential game‐changer in the field.

**Methods:**

Blood samples were collected from the participants and centrifuged to obtain the concentrated growth factor. The poor platelet plasma syringe was placed inside the activated plasma albumin gel device. The ultrastructure of the membrane was examined using a scanning electron microscope (SEM). The weight difference was measured over 21 days to investigate the biodegradability of the samples.

**Results:**

Twenty‐two samples were prepared from six individuals (three males and three females). Based on SEM images, activated albumin gel after 21 days in Hank's solution exhibited a significant decrease in density and evident signs of surface degradation. The weight was significantly reduced after 21 days (*p* < 0.05).

**Conclusion:**

In the present study, the investigation of the ultrastructure and biodegradability of activated albumin gel showed that, based on the observed weight difference, the amount of biodegradation is high, and it may be necessary to use a thicker membrane compared to the conventional thickness of the connective tissue graft.

**Key points:**

- *Enhanced stability and biocompatibility*: The study highlights plasma albumin‐activated gel's potential as a soft tissue scaffold, demonstrating significant biodegradation and structural changes that support cell infiltration and nutrient exchange, essential for tissue regeneration.
- *Controlled degradation profile*: Plasma albumin gel offers a prolonged biodegradation period compared to conventional fibrin membranes, making it suitable for applications requiring stable, long‐lasting scaffolds in soft tissue regeneration.
- *Future clinical applications*: Findings suggest that thicker plasma albumin membranes may be needed for optimal effectiveness, paving the way for further exploration in clinical trials and animal models to validate this approach in soft tissue grafting.

**Plain language summary:**

This study investigates plasma albumin‐activated gel as a promising material for supporting soft tissue repair, particularly in periodontal regeneration. Traditional materials, such as fibrin membranes, are often used to aid healing, but their rapid breakdown can limit effectiveness in the body. Plasma albumin, a protein naturally found in human blood, might offer a more stable alternative by forming a longer‐lasting structure. In this study, researchers processed blood samples from participants to create the gel, examining its structure under a powerful microscope and tracking changes in weight over 21 days to assess its breakdown. Results showed that the gel gradually became less dense and more porous, allowing for cell movement and nutrient flow—both critical for tissue repair. Additionally, a significant reduction in weight indicated a controlled breakdown over time. These findings suggest that plasma albumin‐activated gel may serve as a more durable scaffold for soft tissue regeneration, potentially improving healing outcomes in periodontal applications where a stable, longer‐lasting material is needed.

## INTRODUCTION

The primary considerations of autogenous soft tissue grafts revolve around the discomfort associated with harvesting and the limited tissue availability.^1^, ^2^ Hence, other substances such as allografts and xenografts were introduced; nevertheless, autograft treatment outcomes surpass those of these alternatives.^3^

Platelet concentrates (PCs) have evolved through three main generations, each with distinct characteristics and applications.^4^ First‐generation platelet‐rich plasma releases growth factors quickly, which can stimulate tissue repair and regeneration in various medical fields.^4^, ^5^ Second generation, platelet‐rich fibrin (PRF), which promotes wound healing and soft and hard tissue regeneration.^6^, ^7^, ^8^ Third‐generation advanced PRF—A‐PRF, A‐PRF+, i‐PRF, and T‐PRF—improves mechanical characteristics and biodegradability. Better clinical efficacy of enhanced PRF formulations improves dental procedures requiring tissue regeneration.^9^

Platelet‐ and leukocyte‐rich fibrin have been identified as substances that create a bioactive structure and induce regeneration. As their long‐term stability as a soft tissue substitute is a main concern, plasma albumin gel was introduced as a feasible solution. One of PRF's most significant disadvantages is its lack of long‐term stability in clinical applications,^10^ which makes it absorbable after 10‒14 days of clinical use as a membrane.^11^, ^12^ Increasing the temperature causes albumin to undergo denaturation, resulting in the inactivation of plasmin. In addition to providing a structural framework for cell proliferation, albumin‐enriched biomaterials show a slight reduction over time. Furthermore, it has been shown that association with albumin can modulate the fibrin network's ultrastructure and permeability, producing thicker fibers with a coarse nodular appearance.^13^ This suggests that combining denatured serum albumin with fibrin may enhance the material's stability and biocompatibility, resulting in a durable, autologous material with prolonged efficacy.^13^, ^14^, ^15^ The stability of albumin gel and its structure allow the penetration of cells and biological structures to act as an autologous replacement in the soft tissue of augmentation. Considering that studies related to activated plasma albumin gel (APAG) are limited, this study aims to investigate the ultrastructure and biodegradability of APAG to evaluate its potential to perform as an autogenous soft tissue graft.

## MATERIALS AND METHODS

### Study design

This study was conceptualized as a prospective observational study. The ethical considerations of this study were reviewed and approved by the ethics committee of the Shahid Beheshti Faculty of Dentistry (code of ethics: IR.SBMU.DRC.REC.1400.171). The study was conducted in accordance with the ethical principles of the Declaration of Helsinki.^16^ Informed consent was obtained from all participants. The study adhered to the guidelines for prospective observational studies as outlined by the Strengthening the Reporting of Observational Studies in Epidemiology (STROBE^17^) (Appendix A) checklist to ensure comprehensive and transparent reporting of the research process and findings. Environmental conditions, including temperature (25 ± 1°C), pH 7.4, and humidity (60 ± 5%), were standardized throughout the research period to ensure consistency and reproducibility.

### Study participants

The following inclusion and exclusion criteria were considered for this study:

*Inclusion criteria*: Participants aged 20‒30 years, healthy individuals without systemic diseases, non‐smokers, and not pregnant.
*Exclusion criteria*: Uncontrolled diabetes, immune‐compromised conditions, recreational drug use, smoking, or pregnancy.

The study enrolled systemically healthy individuals between 20 and 30 years of age. Six dental students (three males and three females) meeting the inclusion criteria were voluntarily recruited from Shahid Beheshti University to participate and provide samples. To guarantee participant safety and data integrity, those with uncontrolled diabetes, compromised immune systems, recreational drug use, cigarette smoking, or pregnancy were ineligible.

### Blood sample collection and preparation

The venous blood of the participants was collected. Blood was drawn into 9 mm glass tubes without any additives. These tubes were placed in pairs, facing each other inside a vertical rotor with a fixed‐angle centrifuge (Medifuge, Silfradent), to prepare concentrated growth factor (CGF) in the liquid phase according to the manufacturer's protocol. The centrifugation protocol was as follows: 30 s of acceleration, 2 min at 2700 rpm, 4 min at 2400 rpm, 4 min at 2700 rpm, 3 min at 300 rpm, and 36 s of deceleration, totaling 13 min. This process resulted in the formation of plasma and a layer containing blood cells.^10^, ^12^

### Plasma processing

**FIGURE 1:**
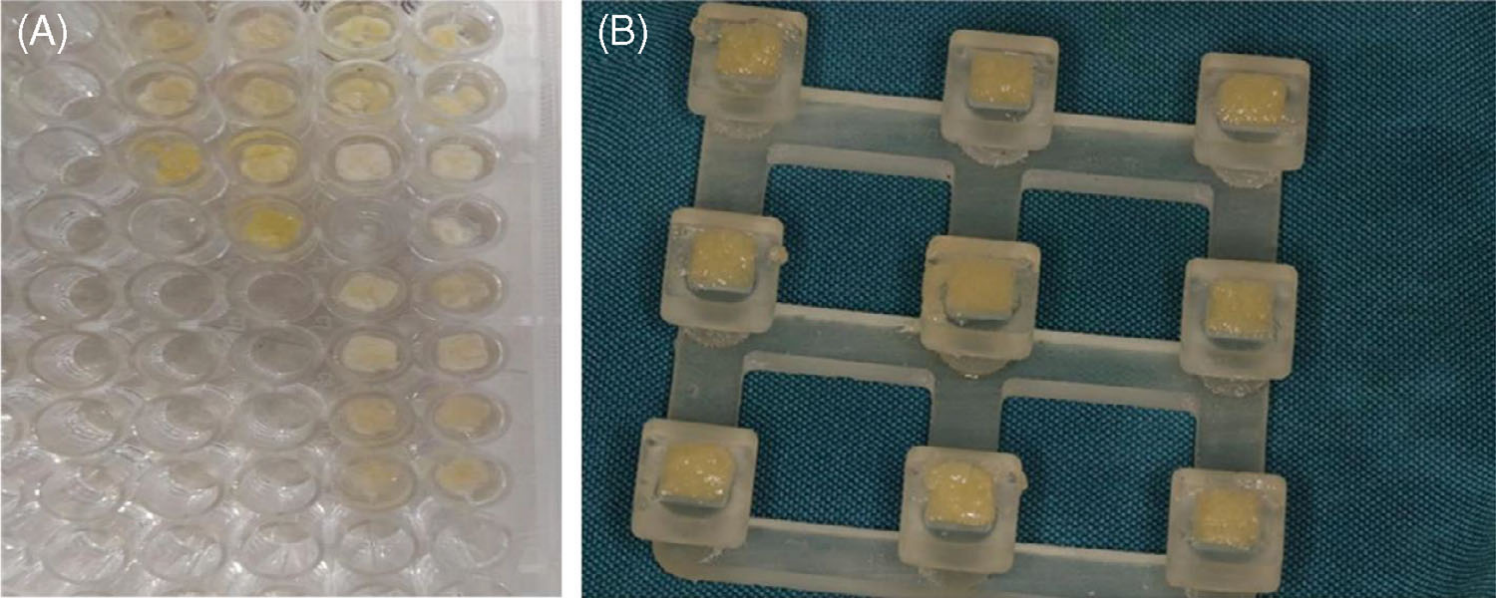
(A) Placing the activated albumin gel in the designed container. (B) The samples were then placed in Hank's solution.

Two milliliters of the upper layer (platelet‐poor plasma) was collected using a 1‐mL syringe. The syringe containing the plasma was placed inside the APAG preparation device (APAG, Silfradent) for 10 min at a temperature of 75°C. Subsequently, the syringes were cooled for 10 min at room temperature, away from light. The plasma was then placed in a container designed for this purpose with a 5 mm length and 2 mm thickness (Figure 1A) (following manufacturer guidelines and Mourão et al.’s protocol^15^).

### Gel fixation and dehydration

To fix the APAG, Karnovsky's solution (4% formaldehyde, 5% glutaraldehyde, and sodium phosphate buffer) was used, followed by treatment with 0.2 M sodium cacodylate and 1% osmium tetroxide solution. Dehydration was performed using a graded series of alcohol (15%‒100%) and hexamethyldecylamine.^13^

### Ultrastructure examination

The ultrastructure of the APAG was examined using a scanning electron microscope (SEM) at 15 kV and magnifications of 5000, 10,000, 25,000, 50,000, 100,000, and 200,000 to investigate the surface morphology of the prepared membrane.^18^ The compactness of the fibrin network, or porosity, was measured directly using SEM and analyzed with ImageJ software (NIH). Porosity was calculated as a percentage by measuring the total pore area (*A*_p_) and the overall image area (*A*_t_). The porosity percentage was then determined using the formula: porosity (%) = (*A*_p_/*A*_t_) × 100. This assessment was crucial for evaluating the internal structure and potential biocompatibility of the APAG. Higher porosity can enhance cell infiltration and nutrient exchange within the scaffold, which is important for the material's application in regenerative medicine.

### Biodegradability assessment

To assess biodegradability, the prepared samples were transferred in a container with ice to the comprehensive research laboratory at Tehran University of Medical Sciences. The initial weight of the samples was recorded after being freeze dried (‒50.5°C, 0.105 mbar). The samples were then placed in Hank's solution for 21 days, with Amphotericin B added to prevent fungal contamination (Figure 1B). Hank's solution was replaced every 3 days. After 21 days, the samples were again freeze dried and weighed. The weight difference was used to evaluate environmental degradation.

### Data and statistical analysis

The information about the samples is presented descriptively. The normality of data distribution was assessed using the Shapiro‒Wilk test. Based on the data distribution, the difference in weight at the beginning and end of the study was evaluated using a *t*‐test, with a significance level of 0.05 was considered. Data analysis was conducted using SPSS software (version 26, SPSS).

## RESULTS

### Sample characteristics

This study sampled six volunteers (three males and three females) with an average age of 23.5 ± 2.5. A total of 22 samples were collected.

### Morphology

**FIGURE 2:**
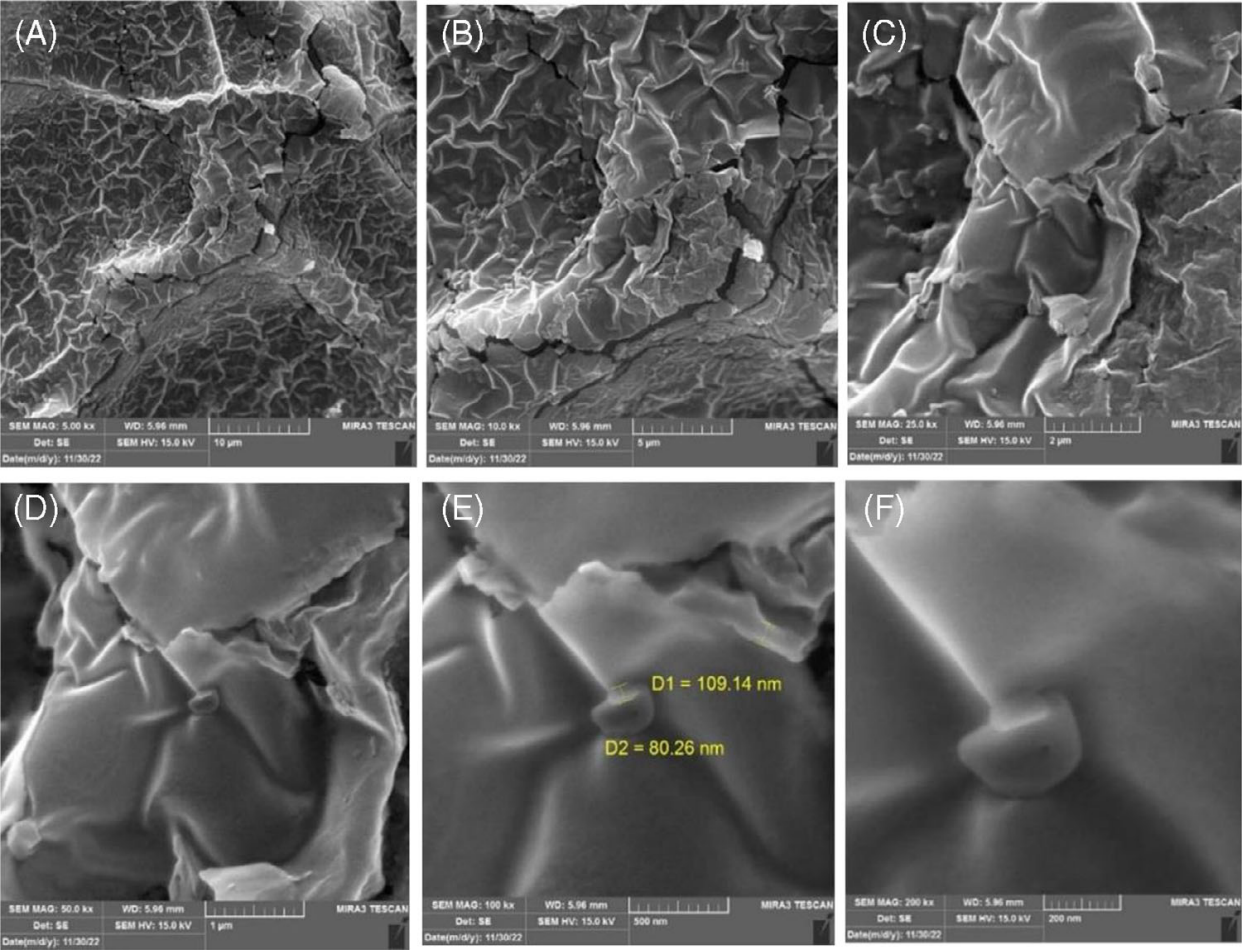
Imaging with scanning electron microscope (SEM) before entering Hank's solution with the following magnifications: (A) 5000×, (B) 10,000×, (C) 25,000×, (D) 50,000×, (E) 100,000×, and (F) 200,000×.

SEM analysis investigated the morphology of the APAG. Figure 2 displays SEM micrographs of the APAG at different magnifications (5000×, 10,000×, 25,000×, 50,000×, 100,000×, and 200,000×) before its introduction to Hank's solution. The initial sample displayed a highly dense surface with a distinct layer of denatured protein deposited on the underlying fibrin fibers. Notably, no cells were observed on this initial surface. Before entering Hank's solution, samples averaged 36.4% porosity. After leaving Hank's solution, porosity reached 54.3% by day 21.

**FIGURE 3:**
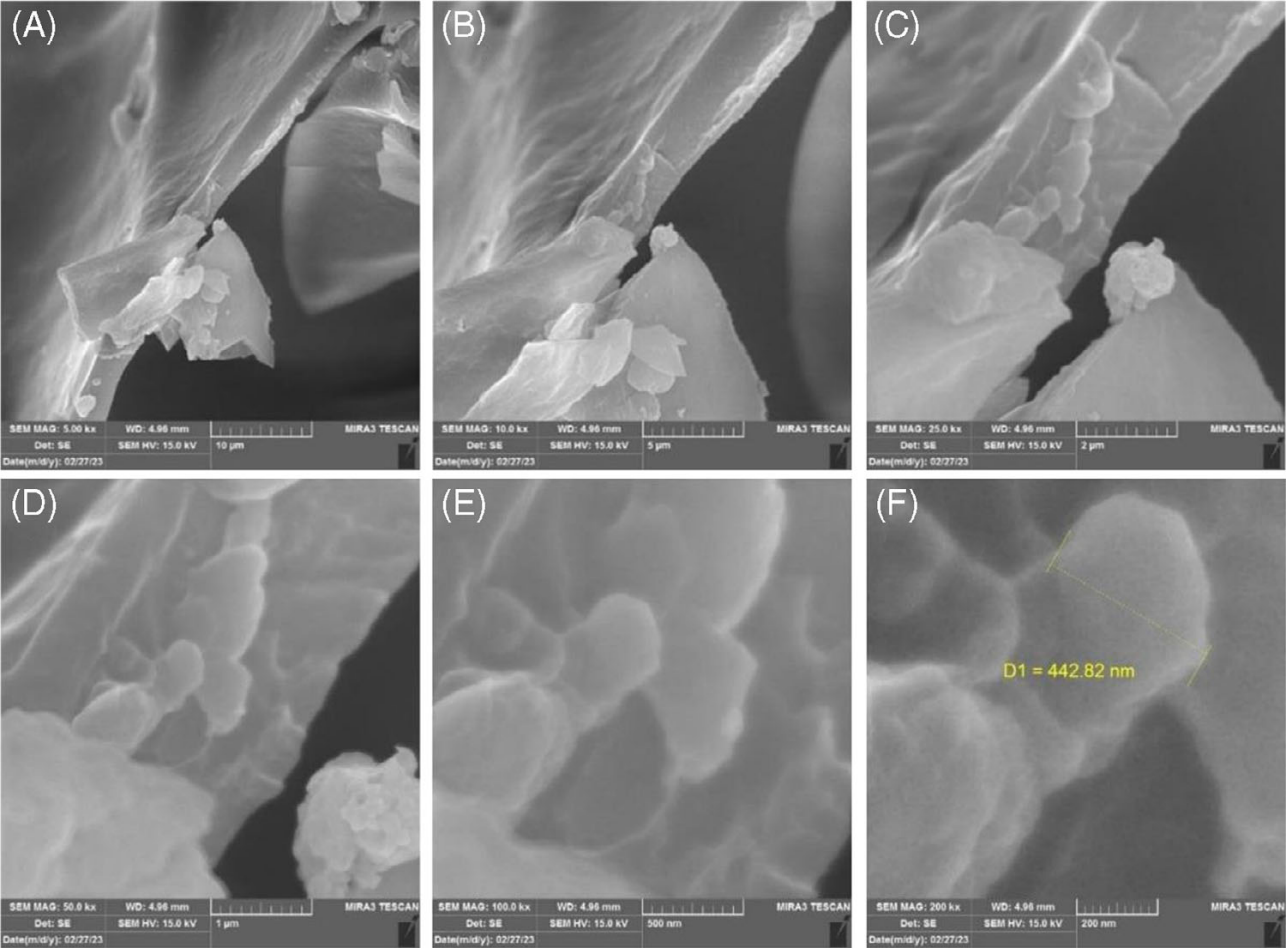
Imaging with scanning electron microscope (SEM) after removing from Hank's solution with magnifications: (A) 5000×, (B) 10,000×, (C) 25,000×, (D) 50,000×, (E) 100,000×, and (F) 200,000×.

In contrast to the initial dense structure, the sample after 21 days in Hank's solution exhibited a significant decrease in density and evident signs of surface degradation (Figure 3). This suggests time‐dependent changes in the gel's properties, possibly due to degradation processes or interactions with the solution.

### Biodegradability assessment

To assess the biodegradability of the APAG, the samples' weight was recorded at two points: before and after immersion in Hank's solution for 21 days. Table 1 summarizes the mean and standard deviation of the sample weights at the beginning and end of the study.

**TABLE 1:** Mean and standard deviation (SD) of samples in biodegradation test.

Groups	Number of samples	Weight of samples (g), mean ± SD	*p*‐value
Group 1			
Baseline	4	0.003 ± 0.0004	0.445
Follow‐up	4	0.002 ± 0.0023	
Group 2			
Baseline	3	0.004 ± 0.0016	0.025
Follow‐up	3	0.002 ± 0.0009	
Group 3			
Baseline	4	0.006 ± 0.0002	0.004
Follow‐up	4	0.002 ± 0.0010	
Group 4			
Baseline	4	0.005 ± 0.0006	0.007
Follow‐up	4	0.003 ± 0.0007	
Group 5			
Baseline	4	0.005 ± 0.0002	0.003
Follow‐up	4	0.002 ± 0.0005	
Group 6			
Baseline	3	0.006 ± 0.0013	0.017
Follow‐up	3	0.003 ± 0.0013	

**FIGURE 4:**
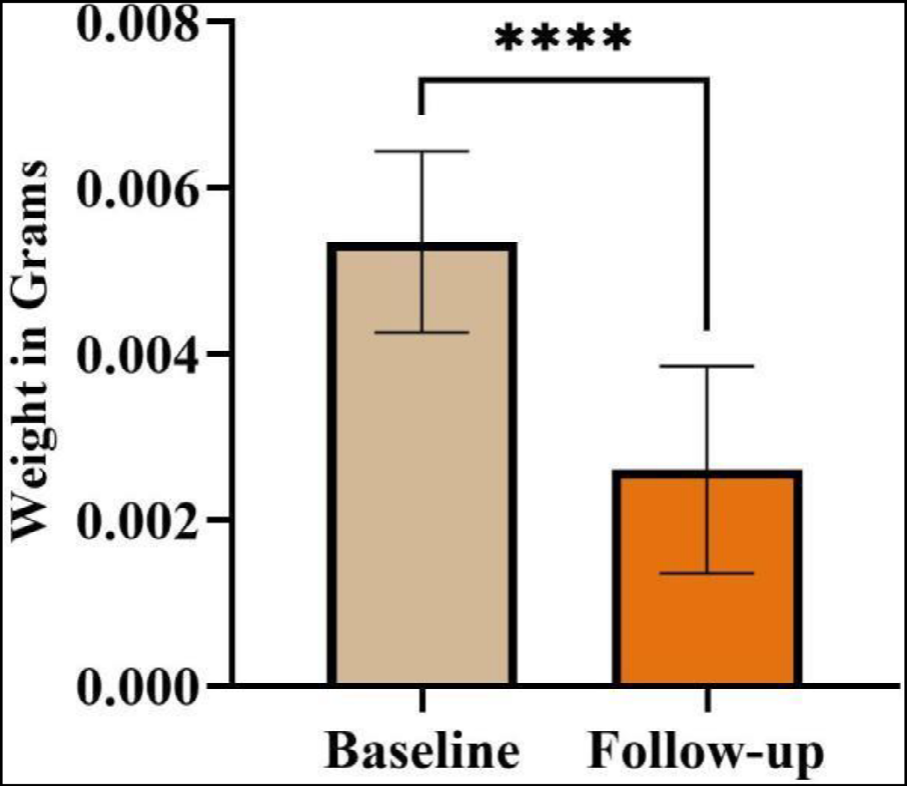
Weight of samples before and after biodegradability test. ^****^Significant *p*‐value.

**FIGURE 5:**
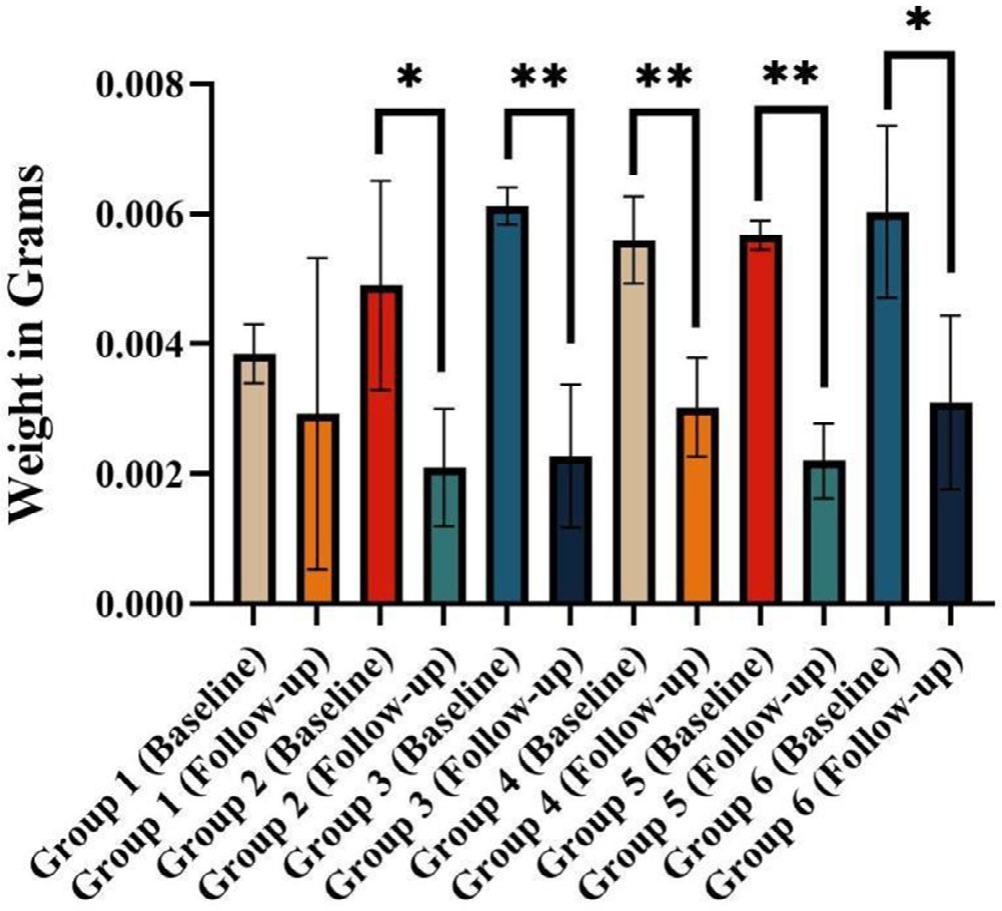
Weight of samples before and after degradability test in each group. ^*^ *p* < 0.05 and ^**^ *p* < 0.01.

The weight measurements provided quantitative data on the biodegradability of the APAG. At the study's outset, the mean weight of the samples was 0.005 ± 0.001 g (95% confidence interval [CI] 0.004‒0.005). This initial weight served as the baseline for biodegradation assessment. Following immersion in Hank's solution for 21 days, a significant decrease in average weight was observed (*p* < 0.0001). The mean weight after 21 days was 0.002 ± 0.001 g (95% CI 0.003‒0.002) (Figure 4). The weight measurements revealed not only a significant decrease in the overall weight of the gel after immersion in Hank's solution (approximately 60%) but also statistically significant weight reductions within individual sample groups (Figure 5).

## DISCUSSION

Soft tissue reconstruction plays an important role in periodontal and peri‐implant health status, patient outcomes, and aesthetics. Connective tissue grafting is considered the gold standard for soft tissue augmentation^1^ and is known for its predictable long‐term stability.^19^, ^20^ Nevertheless, there are drawbacks associated with connective tissue grafts. Harvesting grafts from the palate can lead to morbidity at the donor site^21^ and the amount of available connective tissue is limited, highlighting the need for alternative options.^22^ As a result, various alternative materials from different origins have been introduced to the market.

The findings from this observational study provide preliminary insights into the ultrastructural characteristics and biodegradation behavior of APAG. Based on SEM analysis, the initial structure was dense, potentially limiting cell infiltration. The presence of the denatured protein layer might also have influenced cell adhesion or migration by creating a barrier effect or lacking suitable attachment sites. The gel's characteristics may alter due to degradation or solution interactions. These changes resulted in a decrease in surface density and the degradation of fibrin fibers. The gel became less compact as porosity increased, which may improve scaffold cell penetration and nutrient exchange. This structural shift increases gel biocompatibility and tissue integration, making it a potential scaffold for tissue regeneration.

The SEM findings, including increased porosity and surface degradation over 21 days, suggest enhanced biocompatibility. These structural changes could facilitate better cell infiltration and nutrient exchange, making the material more suitable for clinical applications requiring durable and bioactive scaffolds. The observed degradation and porosity changes indicate a controlled breakdown of the scaffold, which is essential for timely integration with host tissues while preventing premature material failure. Clinically, this balance between stability and biodegradability could ensure prolonged support for tissue regeneration processes, especially in applications where gradual resorption aligns with healing dynamics.

After immersion in Hank's solution, the weight of APAG decreased, suggesting biodegradation. These findings have substantial clinical relevance, indicating that the plasma albumin gel can degrade effectively, and facilitating tissue regeneration without leaving residual material.

APAG offers unique regenerative advantages as it combines albumin denaturation with a slow biodegradation rate, supporting cell adhesion and structural stability over time, which can be beneficial in procedures requiring longer‐lasting scaffolds.^23^ When compared to PRF and other conventional fibrin‐based materials, which typically degrade within 2‒3 weeks, APAG's stability aligns more closely with extended‐PRF methods that last up to 4‒6 months.^24^ This characteristic makes APAG a promising alternative for soft tissue regeneration applications, especially where long‐term membrane function is essential.

Both similarities and discrepancies are revealed when the findings are compared to the existing literature on analogous interventions. The lack of cells on the matrix surface contrasts with Gheno et al.’s investigation,^25^ where the albumin membrane in combination with PRF confined the cells. This discrepancy may be related to the techniques such as not controlling membrane thickness or the short research time. Furthermore, in Mourao's investigation,^15^ the liquid phase of CGF and the Buffy coat were combined, which justifies the presence of cells in the ultrastructural study.

A study in 2014 by Kawase et al., investigated the degradation properties of PRF.^11^ The study indicated a prolonged resorption time (at least 3 weeks) when a heat‐compression technique is applied to PRF. Applying heat to standard PRF membranes through compression significantly enhanced the biodegradability properties of the membranes. Although the study highlighted the stability advantages, a limitation was observed as the cells tended to undergo apoptosis and the growth factor activity decreased during denaturation. Discrepancies between this study and the current one may be due to factors such as membrane thickness control, Hank's solution alteration, the short duration of the study (10 days), and inaccurate measurement of degradation.^11^

It was found in a study by Gheno et al., that the Alb‐PRF membrane exhibited superior biodegradability compared to both L‐PRF and H‐PRF membranes. However, the Alb membrane was obtained using a different protocol, and none of the samples underwent a freeze‐drying process.^25^ Previous research conducted by Kardos et al., in 2018 highlighted the need for improving the performance of PRF due to its rapid degradation and low tensile strength.^26^ To address this, a single‐syringe closed system called hypACT Inject was developed in a study by Kardos et al.,^26^ which produced a sterile and saturable PRF membrane with enhanced handling characteristics using the freeze‒thaw method. The study findings demonstrated significantly higher tensile strength and cell adhesion, as well as a lower degradation rate.

Denatured proteins aggregate mainly through hydrophobic interactions and disulfide bonds, resulting in a biocompatible polymeric material. Albumin does not adhere to leukocytes and platelets because it lacks an integrin‐binding motif such as fibrin's arginyl‐glycyl‐aspartic acid (RGD). Studies suggest that platelets may adhere to folded albumin through receptor‐mediated mechanisms. In addition, it has been suggested that APAG can be mixed with LPCGF in a syringe to produce a mixture containing CD34+ cells and growth factors.^27^

The limitations of this study should also be acknowledged. An extended follow‐up period could offer additional perspectives. Additionally, the small sample size may limit the generalizability of the results, and caution is recommended when interpreting the outcomes of the study. To enhance the accuracy of findings, forthcoming research ought to tackle and mitigate biases like inconsistent heat distribution and issues with sample transfer among different institutions. Subsequent investigations should involve animal models. Furthermore, it is imperative to explore the impact of sample thickness on both stability and biodegradation. Additionally, the feasibility of utilizing albumin gel directly without freeze drying for clinical purposes warrants further examination.

## CONCLUSION

Significant biodegradation was observed through SEM analysis and weight loss measurements. To assess the long‐term effectiveness and durability of the gel, larger trials involving animal models and extended follow‐up periods are necessary. This study highlights the potential of plasma albumin gel as an alternative for soft tissue regeneration, paving the way for further optimization and clinical investigations.

## AUTHOR CONTRIBUTIONS

*Conceptualization, clinical phase, manuscript writing, and final draft approval*: Behzad Houshmand and Mohammadreza Talebi Ardakani. *Data collection, data management, manuscript writing, critical review, and final approval*: Farshad Armandei. *Conceptualization; critical review and final approval*: Anahita Moscowchi, and Hamoun Sabri. *Conceptualization and final approval*: Ahmad Nazari. *Conceptualization, laboratory phase, critical review, and final approval*: Jafar Ai. *Conceptualization, clinical phase, and critical review*: Mehdi Ekhlasmand Kermani.

## CONFLICT OF INTEREST STATEMENT

The authors declare they have no conflicts of interest.

## Data Availability

The data pertinent to this study will be provided upon reasonable request from the corresponding author.
